# Novel Hybrid Formulations Based on Thiourea Derivatives and Core@Shell Fe_3_O_4_@C_18_ Nanostructures for the Development of Antifungal Strategies

**DOI:** 10.3390/nano8010047

**Published:** 2018-01-17

**Authors:** Carmen Limban, Alexandru Vasile Missir, Miron Teodor Caproiu, Alexandru Mihai Grumezescu, Mariana Carmen Chifiriuc, Coralia Bleotu, Luminita Marutescu, Marius Toma Papacocea, Diana Camelia Nuta

**Affiliations:** 1 Department of Pharmaceutical Chemistry, “Carol Davila” University of Medicine and Pharmacy, Traian Vuia No. 6, 020956 Bucharest, Romania; carmen_limban@yahoo.com (C.L.); missir_alexandru@yahoo.com (A.V.M.); diananuta@yahoo.com (D.C.N.); 2 The Organic Chemistry Center of Romanian Academy “Costin D. Nenitescu” Bucharest, Splaiul Independentei, 202B, 77208 Bucharest, Romania; dorucaproiu@gmail.com; 3 Department of Science and Engineering of Oxidic Materials and Nanomaterials, Faculty of Applied Chemistry and Materials Science, University Politehnica of Bucharest, Polizu Street No. 1–7, 011061 Bucharest, Romania; grumezescu@yahoo.com; 4 Department of Microbiology, Faculty of Biology, University of Bucharest, Aleea Portocalelor No. 1–3, 060101 Bucharest, Romania; lumi.marutescu@gmail.com; 5 Research Institute of the University of Bucharest, University of Bucharest, Spl. Independentei 91–95, R-76201 Bucharest, Romania; 6 Stefan Nicolau Institute of Virology, 030304 Bucharest, Romania; cbleotu@yahoo.com; 7 Department of Neurosurgery, “Sf. Pantelimon,” Emergency Hospital, “Carol Davila” University of Medicine and Pharmacy, 021659 Bucharest, Romania; tpapacocea@hotmail.com

**Keywords:** new thiourea derivatives, Fe_3_O_4_@C_18_ nanoparticles, antifungal, *Candida albicans*, biofilm, biocompatibility

## Abstract

The continuously increasing global impact of fungal infections is requiring the rapid development of novel antifungal agents. Due to their multiple pharmacological activities, thiourea derivatives represent privileged candidates for shaping new drugs. We report here the preparation, physico-chemical characterization and bioevaluation of hybrid nanosystems based on new 2-((4-chlorophenoxy)methyl)-*N*-(substituted phenylcarbamo-thioyl)benzamides and Fe_3_O_4_@C_18_ core@shell nanoparticles. The new benzamides were prepared by an efficient method, then their structure was confirmed by spectral studies and elemental analysis and they were further loaded on Fe_3_O_4_@C_18_ nanostructures. Both the obtained benzamides and the resulting hybrid nanosystems were tested for their efficiency against planktonic and adherent fungal cells, as well as for their in vitro biocompatibility, using mesenchymal cells. The antibiofilm activity of the obtained benzamides was dependent on the position and nature of substituents, demonstrating that structure modulation could be a very useful approach to enhance their antimicrobial properties. The hybrid nanosystems have shown an increased efficiency in preventing the development of *Candida albicans* (*C. albicans*) biofilms and moreover, they exhibited a good biocompatibility, suggesting that Fe_3_O_4_@C_18_core@shell nanoparticles could represent promising nanocarriers for antifungal substances, paving the way to the development of novel effective strategies with prophylactic and therapeutic value for fighting biofilm associated *C. albicans* infections.

## 1. Introduction

Fungal infections represent an emerging major world health problem due to the relative paucity and the rapid development of resistance to the existing antifungal drugs [1]. The insertion of the prosthetic medical devices for different exploratory or therapeutic purposes, especially in severe pathological conditions, represents a risk factor for the occurrence of biofilm associated infections. Biofilm’s cells are less susceptible to adverse environmental conditions or stress factors, the infections with microbial pathogens growing in biofilms being very different as compared to those determined by planktonic cells, due to their different behavior, generation time and susceptibility to antimicrobial agents. Different members of the *Candida* genus (particularly *C. albicans* and *C. parapsilosis*) are among the most frequently reported microbial species involved in biofilm associated infections [2]. Biofilms formed by *Candida* sp. cells are associated with drastically enhanced resistance against most antifungal current therapies [3,4]. The expression of drug efflux pumps during the early phase of biofilm formation and alterations in membrane sterols composition contribute to resistance of these biofilms to different antifungal agents [5]. In order to overcome this challenging problem, there is an urgent need for finding and testing new antifungal agents for therapeutic and prophylactic use, both effective on planktonic and biofilm-embedded cells [6].

One of the approaches for the development of novel and effective antimicrobial drugs is to obtain hybrid molecules through the combination of different pharmacophores in one single structure. In this regard, the present research focuses on the preparation, physico-chemical characterization and the evaluation of antibiofilm activity of hybrid nanosystems based on new 2-((4-chlorophenoxy)methyl)-*N*-(substituted phenylcarbamo-thioyl)benzamides and Fe_3_O_4_@C_18_ core@shell nanoparticles against *C. albicans* strains for the development of an efficient strategy for preventing and fighting fungal biofilms. Our hypothesis was formulated taking into account previous research studies highlighting that magnetite nanoparticles could exhibit antimicrobial activity and act synergistically with other antimicrobial substances, or could be successfully used as nanocarriers or for the controlled/prolonged/targeted release of different antimicrobial agents [7,8]. The C_18_ was used as a spacer for facilitating the interaction between the magnetite nanoparticles and the thiourea derivatives and as an easy way to create an interaction between magnetite nanoparticles and thiourea derivatives.

Thiourea derivatives are a class of privileged compounds, exhibiting a wide range of biological activities, such as antibacterial [9], antifungal [10], antituberculosis [11], antiviral [12], anticancer [13], anti-parasitic [14], anticonvulsant [15], anti-oxidant [16], analgesic [17] and anti-inflammatory [18]. Thiourea derivatives also display good coordination ability, being used as intermediates for a great variety of heterocyclic products, such as thiohydantoin [19], iminothiazolidinone [20], thioxopyrimidindione, 1,2,4-thiadiazoles [19], 2*H*-1,2,4-thiadiazolo[2,3-*a*]pyrimidine [21] etc. Novel thiourea derivatives of diphenylphosphoramidate have been synthesized using 4,4′-sulfonyldianiline or 4-aminoaniline, diphenylchlorophosphate and a various aromatic substituted isothiocyanate in the presence of triethylamine. These compounds showed antibacterial activity against *S. aureus*, *B. subtilis*, *P. aeruginosa* and *E. coli* strains better—as well as antifungal activity on *Trichoderma viridae* and *Aspergillus niger*—than the standard ciprofloxacin drug and miconazole, respectively [22]. Acetyl, chloroacetyl and benzoyl thiourea derivatives of carboxymethyl chitosan proved superior antimicrobial activity against *B. subtilis*, *S. aureus* and *E. coli* bacterial strains and the pathogenic fungi *A. fumigatus*, *Geotrichumcandidum* and *Candida albicans.* Acylthiourea derivatives of carboxymethyl chitosan exhibited a higher antimicrobial activity especially against Gram-positive bacteria and significant antifungal activity, shown mainly by the chloroacetyl derivatives [23]. On the other side, magnetite nanoparticles were frequently reported in different studies to exhibit a great potential to modulate microbial biofilms. However, till now little is known about the intimated mechanisms of its anti-biofilm activity [24,25,26].

## 2. Results

**Scheme 1:**
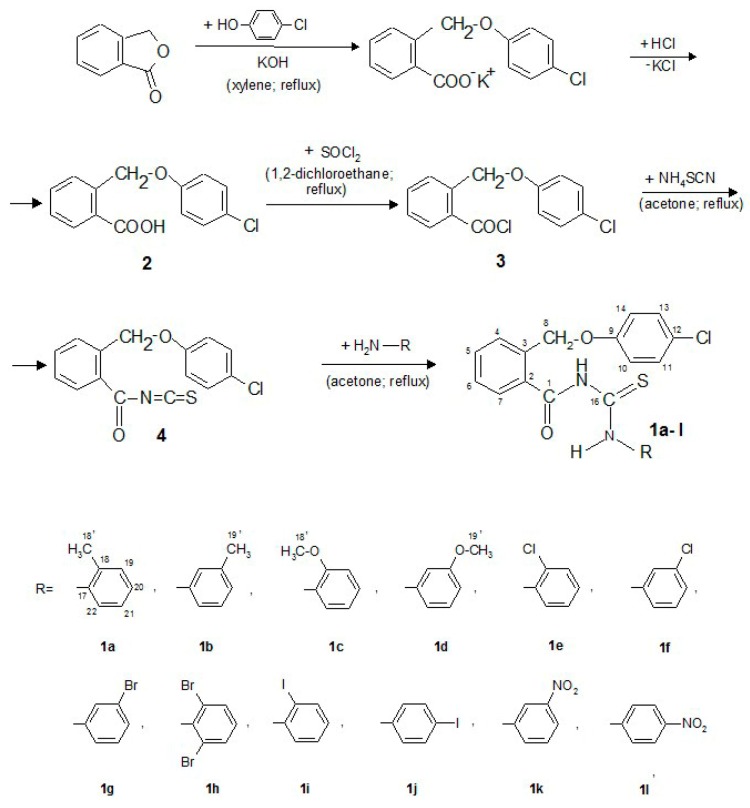
Synthetic pathway for the new *N*-phenylcarbamothioylbenzamides**1a–l**.

The target compounds (**1a–l**) were synthesized by a series of reactions as shown in Scheme 1. The new derivatives are white or yellow crystalline solids, soluble at room temperature in acetone and chloroform, by heating in lower alcohols, benzene, toluene and xylene and insoluble in water.

The infrared absorption (IR) bands were given as w—weak, m—medium, s—strong, vs—very strong.

The structures of the new compounds were also determined from their nuclear magnetic resonance (NMR) spectra. The new thiourea derivatives were dissolved in DMSO-d6 (hexadeuteriodimethyl sulphoxide) and the chemical shifts values, expressed in parts per million (ppm) were referenced downfield to tetramethylsilane, for ^1^H-NMR and ^13^C-NMR and the constants (*J*) values in Hertz.

The chemical shifts for hydrogen and carbon atoms were established also by gradient-selected absolute value correlation (gCOSY), gradient-selected heteronuclear multiple bond correlation (gHMBC), gradient selected heteronuclear single-quantum correlation (gHSQC)–DNMR experiments.

The ^1^H-NMR data are reported in the following order: chemical shifts, multiplicity, the coupling constants, number of protons and signal/atom attribution. The apparent resonance multiplicity is described as s (singlet), d (doublet), t (triplet), m (multiplet), dd (double doublet), dt (double triplet), td (triple doublet), ddd (doublet of double doublets) and br (broad) signal.

For the ^13^C-NMR data the order is the following: chemical shifts and signal/atom attribution (Cq-quaternary carbon).

The XRD (X-Ray Diffraction) pattern of the fabricated Fe_3_O_4_@C_18_ nanoparticles show that all diffraction peaks can be easily indexed to be a pure cubic structure by the characteristic peaks [2θ = 30.2° (220), 35.5°(311), 43.2° (400), 54.5° (422), 57.1° (511), 62.7° (440), which matches well with the reported value, indicating the formation of Fe_3_O_4_ phase.

The IR analysis identified the C_18_ organic shell on the surface of fabricated core@shell nanostructures. Two sharp bands at 2915 and 2848 cm^−1^ were attributed to the asymmetric –CH_2_– stretching and the symmetric –CH_2_– stretching, respectively. The peak recorded at about 1701 cm^−1^ at IR spectrum of the fabricated core@shell nanostructures showed the C=O stretching vibration of C_18_.

The size of fabricated core@shell nanostructure not exceeding 15 nm and their spherical shape were confirmed by TEM (Transmission Electron Microscopy) analysis according to our previous published results [27].

**Figure 1:**
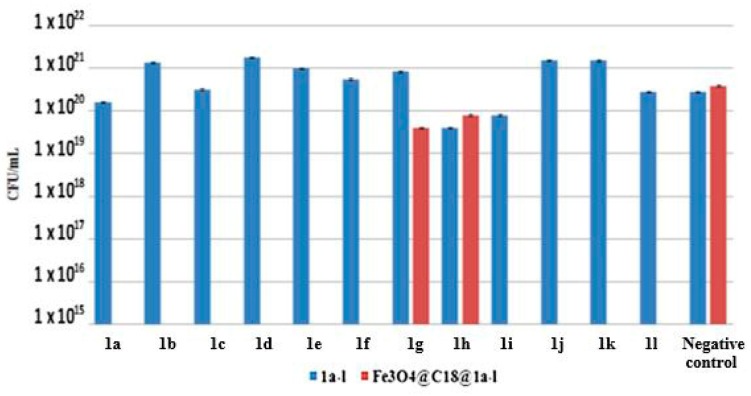
Viable cell counts of fungal cells harvested from 24 h biofilms developed on glass slides covered with bare compounds (C) (blue) or hybrid nanosystems (thiourea derivatives loaded at the same concentration in nanoparticles) (red).

The microbiological assays results are demonstrating that out of the 12 tested compounds, only two, i.e., **1h** and **1i** proved to slightly inhibit the development of *C. albicans* biofilms at 24 h, when they were pelliculised on the glass slide support. In exchange, when incorporated into nanoparticles, the majority of the tested compounds (excepting **1g** and **1h**) proved to prevent efficiently the biofilm development on the functionalized surfaces, as compared with the negative control, represented by the glass slide pelliculised only with nanoparticles (Figure 1).

**Figure 2:**
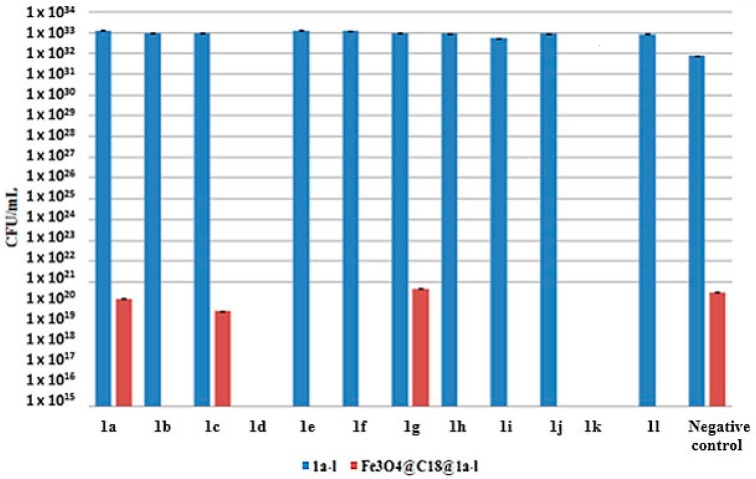
Viable cell counts of fungal cells harvested from 48 h biofilms developed on glass slides covered with on glass slides covered with bare compounds (C) (blue) or hybrid nanosystems (thiourea derivatives loaded at the same concentration in nanoparticles) (red).

The antibiofilm effect of the obtained nanosystem was also maintained at 48 h, the microbial biofilm development being totally inhibited by the majority of the tested compounds, excepting **1a**, **1c** and **1g**. The compounds **1a** and **1c** were active against the 24 h biofilm but could not inhibit the fungal biofilm development at 48 h (Figure 2).

**Figure 3:**
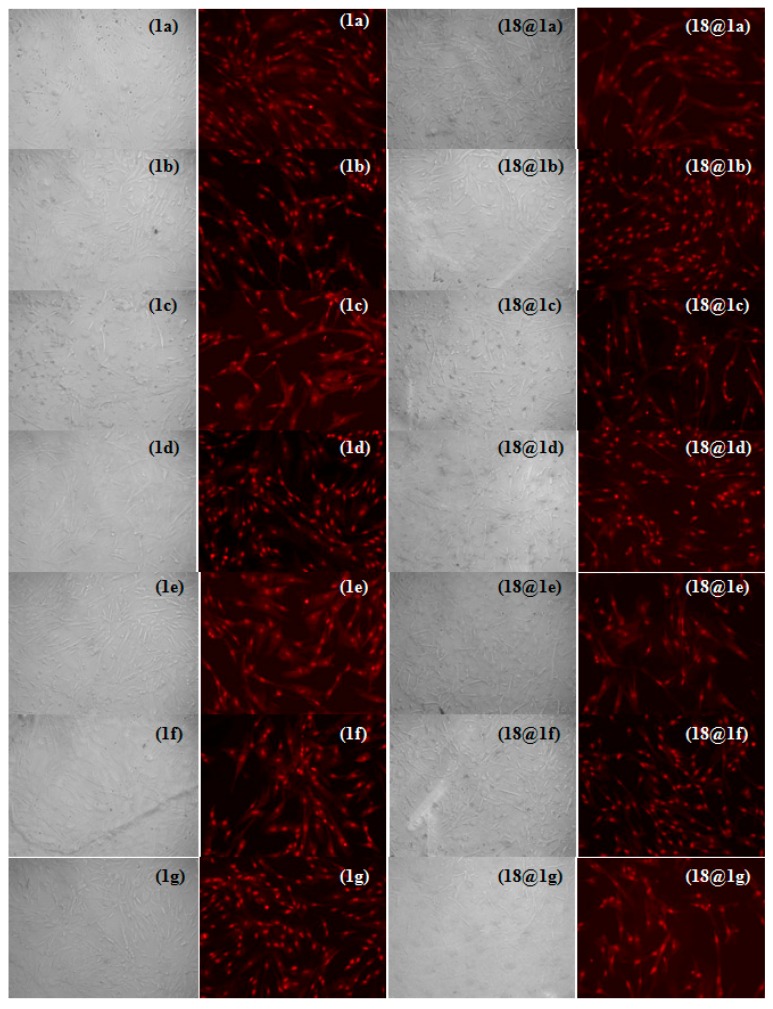

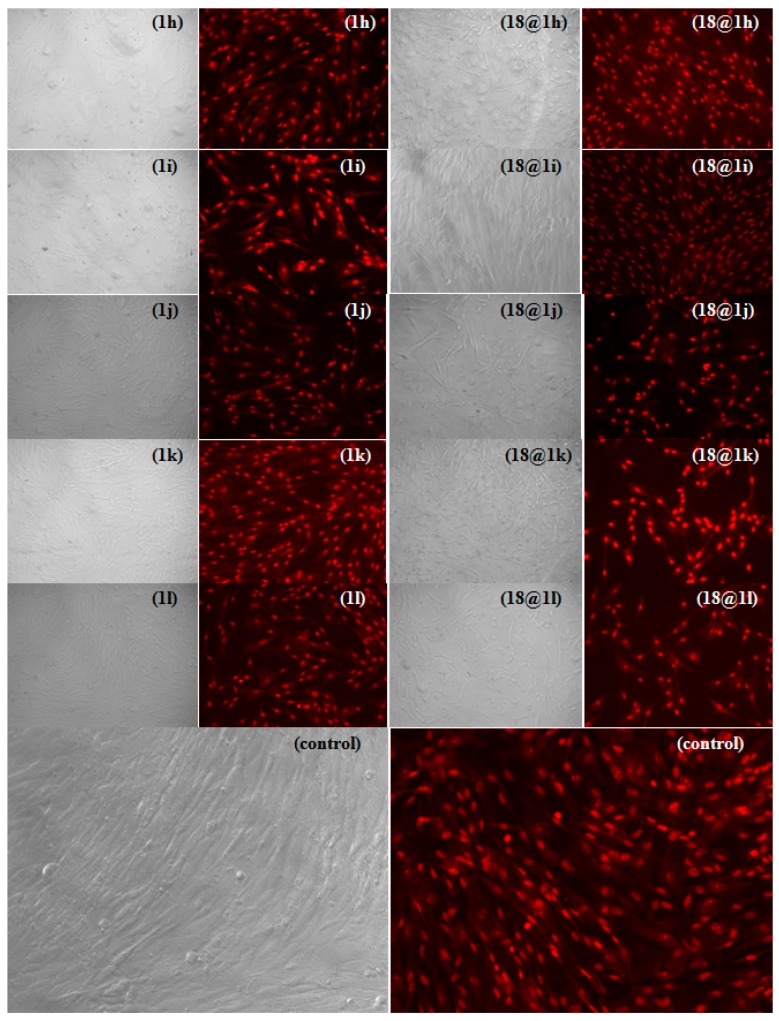
Black/white and fluorescence inverted microscopy images of the mesenchymal cells adhered for 24 h on: microscopic slides (control), microscopic slides coated with the bare compounds (**1a–l**) and respectively with thiourea derivatives loaded in the same concentration in Fe_3_O_4_@C_18_ nanoparticles (18@**1a–l**) (200×).

The in vitro study of the interaction of the hybrid nanosystems with the mesenchymal stem cells, showing that the slides covered with bare compounds or with hybrid nanosystems (represented by thiourea derivatives loaded at the same concentration in nanoparticles) allowed the adherence and proliferation of the eukaryotic cells, in a similar manner to that observed on the microscopic slides control (Figure 3).

## 3. Discussion

*C. albicans* is the major human fungal pathogen causing a variety of clinical infections, ranging from superficial mucosal diseases to deep mycoses, being usually associated with biofilm development on medical devices [28]. After a catheter, or other implanted device, is inserted into the body, the surface of the biomaterial is rapidly covered by a conditioning film which alters the surface properties of the biomaterial. The majority of the molecules are proteinaceous, such as fibronectin, fibrinogen and fibrin which have been shown to enhance the adherence of Gram-positive cocci, Gram-negative rods and *C. albicans*. The fungal biofilms developed on different substrata, such as cellulose, poly-styrene, silicone, polyurethane and acrylate proved to exhibit multifactorial resistance mechanisms to many classes of antifungal agents [29]. Resistance of *Candida* biofilms to antifungal agents was first demonstrated in 1995 [30]. All clinically important antifungal agents—amphotericin B, azoles, flucytosine, proved to be much less active against *C. albicans* biofilms developed on polyvinyl chloride (PVC) discs than against planktonic cells and drug concentrations required to reduce metabolic activity by 50% were five to eight times higher for biofilms and 30–2000 times higher than the corresponding minimum inhibitory concentrations (MICs).

These findings are clearly demonstrating the necessity of finding new strategies for fighting fungal biofilm-associated infections. Two main research directions have been approached, i.e.,: (a) development of biomaterials with anti-adhesive properties using physico-chemicals methods and (b) incorporation in or coating current biomaterials with bioactive antifungal compounds [31]. In the present study, we addressed the second approach, referring to obtaining a functionalized surface, pelliculised with a nanolayer consisting of ferrite nanoparticles loaded with the newly synthesized 2-((4-chlorophenoxy)methyl)-*N*-(substituted phenylcarbamothioyl)benzamides, exhibiting antifungal activity.

The chemical structures of the new compounds were characterized by their melting point, elemental analysis, infrared and NMR spectral studies. The spectral data and elemental analysis data of the new compounds were in agreement with the proposed structures. The obtained compounds proved to successfully inhibit the early fungal biofilms, quantified after 24 h. The less efficiency of the tested derivatives against the 48 h biofilms could be explained by the complex structure of these mature biofilms, consisting of a dense network of yeasts, hyphae and pseudohyphae, as demonstrated by previous in vitro studies using plastic surfaces and catheter samples [32]. In contrast with the 24 h fungal biofilm—which is thinner and consists mainly of yeast cells that are probably more susceptible to the active compound—the 48 h biofilm is thicker, preventing the accumulation of the active substances inside biofilm.

Our previous studies have demonstrated that magnetite nanoparticles could be used for the design of hybrid nanostructures that improve the antimicrobial activity of chemical substances and essential oils. Our results are demonstrating that the combination between nanoparticles and certain thiourea derivatives could act as efficient antibiofilm agents. However, to confirm the medical utility of these combinations, in vivo studies and clinical trials are further needed.

The derivatives, having as substituents *metha*-methyl and *metha*-methoxy, iodine, chlorine and nitro in different positions, proved the same antibiofilm efficiency when loaded into nanoparticles. The less active compounds were **1a**, **1c**, **1g** and **1h**, having as substituents *ortho*-methyl, *ortho*-methoxy, *metha*-bromide, 1, 6-di-bromide. Our results show that the substitution in *metha* position with methyl and methoxy groups, is favorable for the antibiofilm effect as compared with that in *ortho* for the same functional groups. The substitution with bromide, irrespective to the position or number of the substituent groups is not favorable for the occurrence of an antibiofilm effect.

The promising anti-adherence properties of the obtained nanosystems are enhanced by their increased in vitro biocompatibility, assessed on human mesenchymal cells.

The obtained results are clearly demonstrating that the magnetic nanoparticles are improving the antifungal effect of the new thiourea derivatives, probably by modifying their functional groups and by increasing the volume surface ratio and thus the contact between the active compound and microbial target.

## 4. Materials and Methods

### 4.1. Materials

The chemicals for the synthesis of the new thioureides were purchased from Merck (Hohenbrunn, Germany), and Sigma-Aldrich (Steinheim, Germany) companies and used as such without further purification except acetone which was dried over K_2_CO_3_ and then distilled and ammonium thiocyanate which was treated by heating at 100 °C before use.

### 4.2. Synthesis and Spectral Characterization of Adsorption-Shell

Melting points were determined with an Electrothermal 9100 (Bibby Scientific Ltd., Stone, UK) capillary melting point apparatus in open capillary tubes and are uncorrected.

C, H, N and S analysis were carried out on a Perkin Elmer CHNS/O Analyzer Series II 2400 elemental analyzer (PerkinElmer Instruments, Shelton, CT, USA).

The room temperature attenuated total reflection Fourier transform infrared (FT-IR ATR) spectra of the all synthesized compounds were registered using a Bruker Vertex 70 spectrophotometer (Bruker Corporation, Billerica, MA, USA).

All NMR spectra were recorded on a Varian Unity Inova 400 instrument (Varian Inc., Palo Alto, CA, USA) operating at 400 MHz for ^1^H and 100 MHz for ^13^C.

The 2-(4-chlorophenoxymethyl)benzoic acid (**2**) and 2-(4-chlorophenoxy-methyl)benzoyl chloride (**3**) derivatives were prepared in good yields according to the previous article [33,34].

#### 4.2.1. General Synthesis Procedure of the New Thioureides

The compounds **1a–l** were prepared by a following procedure. A solution of 2-(4-chlorophenoxymethyl)benzoyl chloride (**3**) (0.01 mol) in acetone (15 mL) was added to a solution of ammonium thiocyanate (0.01 mol) in acetone (5mL) to afford arylisothiocyanate (**4**) in situ. The reaction mixture was heated under reflux for 1 h and then cooled at the room temperature. A solution of primary amine (0.01 mol) in acetone (2 mL) was added to the mixture and heated under reflux for 1 h. The acylthioureas were precipitated after the cooled reaction mixture was poured into 500 mL water. The solid product was purified by recrystallization from isopropanol with active carbon.

*2-((4-Chlorophenoxy)methyl)-N-(2-methylphenylcarbamothioyl)benzamide* (**1a**). Yield 68%; melting temperature (mp) 147.4–149 °C; ^1^H-NMR (DMSO-d6): 12.11 (br s, 1H, NH, deuterable); 11.88 (br s, 1H, NH, deuterable); 7.62 (bd, *J* = 7.2 Hz, 1H, H-7); 7.59 (bd, *J* = 7.5 Hz, 1H, H-4); 7.57 (td, *J* = 1.4 Hz, *J* = 7.5 Hz, 1H, H-5); 7.49 (td, *J* = 1.4 Hz, *J* = 7.5 Hz, 1H, H-6); 7.47 (m, 1H, H-22); 7.33–7.18 (m, 3H, H-19, H-20, H-21); 7.31 (d, *J* = 9.0 Hz, 2H, H-11, H-13); 7.01 (d, *J* = 9.0 Hz, 2H, H-10, H-14); 5.31 (s, 2H, H-8); 2.13 (s, 3H, H-18’); ^13^C-NMR (DMSO-d6): 179.76 (C-16); 170.08 (C-1); 156.97 (C-9); 136.68 (Cq); 135.04 (Cq); 133.44 (Cq); 133.20 (Cq); 130.90; 130.28; 129.17 (C-11, C-13); 128.57; 128.49; 127.90; 126.91; 126.42; 125.97; 124.62 (C-12); 116.34 (C-10, C-14); 67.81 (C-8); 17.33 (C-18’). FT-IR (solid in ATR, ν cm^−1^): 3163m; 3057w; 2864w; 1682s; 1594w; 1509vs; 1488vs; 1460m; 1383w; 1329w; 1238s; 1195w; 1168m; 1150m; 1089w; 1029w; 1005m; 849w; 767m; 676w; 655m. Anal. Calcd for C_22_H_19_ClN_2_O_2_S (410.91): C, 64.31; H, 4.66; Cl, 8.63; N, 6.82; S, 7.8%; Found: C, 64.61; H, 4.76; Cl, 8.71; N, 6.80; S, 7.84%.

*2-((4-Chlorophenoxy)methyl)-N-(3-methylphenylcarbamothioyl)benzamide* (**1b**). Yield 68%; mp 126.9–128.6 °C; ^1^H-NMR (DMSO-d6): 12.37 (br s, 1H, NH, deuterable); 11.82 (br s, 1H, NH, deuterable); 7.62 (bd, *J* = 7.2 Hz, 1H, H-7); 7.59 (bd, *J* = 7.5 Hz, 1H, H-4); 7.57 (td, *J* = 1.4 Hz, *J* = 7.51 Hz, H, H-5); 7.48 (td, *J* = 1.4 Hz, *J* = 7.5 Hz, 1H, H-6); 7.43 (bd, *J* = 8.6 Hz, 1H, H-22); 7.32 (bs, 1H, H-10); 7.29 (t, *J* = 7.8 Hz, 1H, H-21); 7.08 (bd, *J* = 7.8 Hz, 1H, H-20); 7.31 (d, *J* = 9.0 Hz, 2H, H-11, H-13); 7.01 (d, *J* = 9.0 Hz, 2H, H-10, H-14); 5.31 (s, 2H, H-8); 2.33 (s, 3H, H-19’). ^13^C-NMR (DMSO-d6): 178.75 (C-16); 170.04 (C-1); 156.96 (C-9); 137.99 (Cq); 137.63 (Cq); 135.15 (Cq); 133.28 (Cq); 130.96; 129.16 (C-11, C-13); 128.46; 128.36; 128.34; 127.83; 126.87; 124.54 (C-12); 124.48; 121.26; 116.36 (C-10, C-14); 67.75 (C-8); 20.80 (C-19’). FT-IR (solid in ATR, ν cm^−1^): 3158m; 3024m; 2884w; 1680s; 1596m; 1528vs; 1487s; 1379m; 1325m; 1275m; 1240s; 1174m; 1151s; 1083m; 1003m; 825m; 789w; 773w; 747m; 720m; 692w; 668w; 643w. Anal. Calcd for C_22_H_19_ClN_2_O_2_S (410.91): C, 64.31; H, 4.66; Cl, 8.63; N, 6.82; S, 7.8%; Found: C, 64.09; H, 4.61; Cl, 8.72; N, 6.87; S, 7.76%.

*2-((4-Chlorophenoxy)methyl)-N-(2-methoxyphenylcarbamothioyl)benzamide* (**1c**). Yield 64%; mp 128.4–130.6 °C; ^1^H-NMR (DMSO-d6): 12.74 (br s, 1H, NH, deuterable); 11.82 (br s, 1H, NH, deuterable); 8.51 (dd, *J* = 1.4 Hz, *J* = 8.2 Hz, 1H, H-22); 7.65 (bd, *J* = 7.4 Hz, 1H, H-7); 7.60 (bd, *J* = 7.4 Hz, 1H, H-4); 7.59 (td, *J* = 1.4 Hz, *J* = 7.5 Hz, 1H, H-5); 7.49 (td, *J* = 1.4 Hz, *J* = 7.5 Hz, 1H, H-6); 7.29 (d, *J* = 9.0 Hz, 2H, H-11, H-13); 7.23 (td, *J* = 8.2 Hz, *J* = 1.4 Hz, 1H, H-20); 7.12 (dd, *J* = 1.4 Hz, *J* = 8.2 Hz, 1H, H-19); 7.02 (d, *J* = 9.0 Hz, 2H, H-10, H-14); 7.01 (td, *J* = 8.2 Hz, *J* = 1.4 Hz, 1H, H-21); 5.34 (s, 2H, H-8); 3.96 (s, 3H, H-18’). ^13^C-NMR (DMSO-d6): 177.64 (C-16); 169.99 (C-1); 156.90 (C-9); 150.42 (C-18); 135.03 (Cq); 133.34 (Cq); 130.92; 129.07 (C-11, C-13); 128.54; 128.48; 127.87; 126.67 (C-17); 126.48; 124.58 (C-12); 123.03; 119.61 (C-19); 116.38 (C-10, C-14); 111.18 (C-19); 67.86 (C-8); 55.84 (C-18’). FT-IR (solid in ATR, ν cm^−1^): 3288w; 3002w; 2937w;2837w; 1667w; 1601s; 1554vs;1529vs; 1486s; 1463m; 1356s; 1325m; 1287m; 1241vs; 1222m; 1181m; 1151m; 1051w; 1027s; 818m; 794w; 735m; 692w; 665w. Anal. Calcd for C_22_H_19_ClN_2_O_3_S (426.91): C, 61.90; H, 4.49; Cl, 8.30; N, 6.56; S, 7.51%; Found: C, 61.69; H, 4.36; Cl, 8.21; N, 6.58; S, 7.54%.

*2-((4-Chlorophenoxy)methyl)-N-(3-methoxyphenylcarbamothioyl)benzamide* (**1d**). Yield 67%; mp 133–135.2 °C; ^1^H-NMR (DMSO-d6): 12.42 (br s, 1H, NH, deuterable); 11.83 (br s, 1H, NH, deuterable); 7.63 (bd, *J* = 7.2 Hz, 1H, H-7); 7.59 (bd, *J* = 7.4 Hz, 1H, H-4); 7.57 (td, *J* = 1.4 Hz, *J* = 7.5 Hz, 1H, H-5); 7.48 (td, *J* = 1.4 Hz, *J* = 7.5 Hz, 1H, H-6); 7.34 (bs, 1H, H-18); 7.31 (t, *J* = 8.2 Hz, 1H, H-21); 7.31 (d, *J* = 9.0 Hz, 2H, H-11, H-13); 7.11 (bd, *J* = 8.2 Hz, 1H, H-22); 7.01 (d, 9.0 Hz, 2H, H-10, H-14); 6.84 (dd, *J* = 2.2 Hz, *J* = 8.2 Hz, 1H, H-20); 5.31 (s, 2H, H-8); 3.77 (s, 3H, H-19’). ^13^C-NMR (DMSO-d6): 178.62 (C-16); 169.97 (C-1); 159.21 (C-19); 156.94 (C-9); 138.81 (Cq); 135.18 (Cq); 133.20 (Cq); 131.00; 129.35; 129.15 (C-11, C-13); 128.50; 128.33; 127.81; 124.61 (C-12); 116.39 (C-10, C-14); 116.19; 111.84; 109.52; 67.71 (C-8); 55.12 (C-19’). FT-IR (solid in ATR, ν cm^−1^): 3293w; 3056w; 2998w; 2955w; 2916w; 2832w; 1671m; 1597m; 1570m; 1528vs; 1489s; 1469m; 1380m; 1355m; 1330m; 1277s; 1239vs; 1154s; 1121m; 1102m; 1032m; 1005w; 813m; 779w; 756w; 689m; 647w. Anal. Calcd for C_22_H_19_ClN_2_O_3_S (426.91): C, 61.90; H, 4.49; Cl, 8.30; N, 6.56; S, 7.51%; Found: C, 62.07; H, 4.56; Cl, 8.41; N, 6.51; S, 7.62%.

*2-((4-Chlorophenoxy)methyl)-N-(2-chlorophenylcarbamothioyl)benzamide* (**1e**). Yield 76%; mp 124.3–125.7 °C; ^1^H-NMR (DMSO-d6): 12.47 (br s, 1H, NH, deuterable); 12.06 (br s, 1H, NH, deuterable); 7.96 (dd, *J* =1.6 Hz, *J* =7.6 Hz, 1H, H-19); 7.65 (bd, *J* = 7.4 Hz, 1H, H-7); 7.60–7.55 (m, 3H, H-4, H-5, H-22); 7.49 (td, *J* = 1.4 Hz, *J* = 7.5 Hz, 1H, H-6); 7.40 (td, *J* = 7.6 Hz, *J* = 1.6 Hz, 1H, H-20); 7.32 (td, *J* = 7.6 Hz, *J* = 1.6 Hz, 1H, H-21); 7.30 (d, *J* = 9.0 Hz, 2H, H-11, H-13); 7.01 (d, *J* = 9.0 Hz, 2H, H-10, H-14); 5.31 (s, 2H, H-8). ^13^C-NMR (DMSO-d6): 179.89 (C-16); 170.21 (C-1); 156.91 (C-9); 135.13 (Cq); 133.19 (Cq); 131.03; 129.37; 129.12 (C-11, C-13); 128.58; 128.56; 128.01; 127.92; 127.73; 127.08; 124.60 (C-12); 116.35 (C-10, C-14); 67.80 (C-8). FT-IR (solid in ATR, ν cm^−1^): 3247m; 3031w; 2923w; 1675m; 1582m; 1527vs; 1491vs; 1444m; 1386w; 1325m; 1283m; 1243vs; 1159s; 1095w; 1034m; 815m; 796w; 747m; 728w; 685m; 662w. Anal. Calcd for C_21_H_16_Cl_2_N_2_O_2_S (431.33): C, 58.48; H, 3.74; Cl, 16.44; N, 6.49; S, 7.43%; Found: C, 58.27; H, 3.61; Cl, 16.51; N, 6.51; S, 7.38%.

*2-((4-Chlorophenoxy)methyl)-N-(3-chlorophenylcarbamothioyl)benzamide* (**1f**). Yield 66%; mp 118.1–119.8 °C; ^1^H-NMR (DMSO-d6): 12.40 (br s, 1H, NH, deuterable); 11.92 (br s, 1H, NH, deuterable); 7.81 (t, *J* = 1.8 Hz, 1H, H-18); 7.64 (bd, *J* = 7.2 Hz, 1H, H-7); 7.59 (bd, *J* = 7.5 Hz, 1H, H-4); 7.57 (td, *J* = 1.4 Hz, *J* = 7.5 Hz, 1H, H-5); 7.48 (td, *J* = 1.4 Hz, *J* = 7.5 Hz, 1H, H-6); 7.46 (dt, *J* = 8.0 Hz, *J* = 1.8 Hz, 1H, H-22); 7.43 (t, *J* = 8.0 Hz, 1H, H-21); 7.33 (dt, *J* = 8.0 Hz, *J* = 1.8 Hz, 1H, H-20); 7.31 (d, *J* = 9.0 Hz, 2H, H-11, H-13); 7.01(d, *J* = 9.0 Hz, 2H, H-10, H-14); 5.31 (s, 2H, H-8). ^13^C-NMR (DMSO-d6): 179.17 (C-16); 169.87 (C-1); 156.93 (C-9); 139.20 (Cq); 135.21 (Cq); 133.15 (Cq); 132.56 (Cq); 131.04 (Cq); 130.16; 129.15 (C-11, C-13); 128.49; 128.35; 127.83; 126.04; 124.62 (C-12); 123.98; 123.07; 116.35 (C-10, C-14); 67.69 (C-8). FT-IR (solid in ATR, ν cm^−1^): 3222m; 3105m; 3062m; 3034m; 1666m; 1585m; 1525vs; 1491vs; 1479vs; 1437s; 1413m; 1323m; 1292m; 1237vs; 1153s; 1092m; 1051m; 1025m; 949w; 872w; 814m; 751m; 722m; 704m; 679m; 658m. Anal. Calcd for C_21_H_16_Cl_2_N_2_O_2_S (431.33): C, 58.48; H, 3.74; Cl, 16.44; N, 6.49; S, 7.43%; Found: C, 58.31; H, 3.65; Cl, 16.56; N, 6.45; S, 7.47%.

*2-((4-Chlorophenoxy)methyl)-N-(3-bromophenylcarbamothioyl)benzamide* (**1g**). Yield 73%; mp 126–127.7 °C; ^1^H-NMR (DMSO-d6): 12.39 (br s, 1H, NH, deuterable); 11.92 (br s, 1H, NH, deuterable); 7.93 (t, *J* = 1.2 Hz, 1H, H-18); 7.62 (bd, *J* = 7.2 Hz, 1H, H-7); 7.59 (bd, *J* = 7.5 Hz, 1H, H-4); 7.57 (td, *J* = 1.4 Hz, *J* = 7.5 Hz, 1H, H-5);7.53–7.44 (m, 3H, H-6,H-20, H-22); 7.37 (t, *J* = 8.0 Hz, 1H, H-21); 7.31 (d, *J* = 9.0 Hz, 2H, H-11, H-13); 7.01 (d, *J* = 9.0 Hz, 2H, H-10, H-14); 5.31 (s, 2H, H-8). ^13^C-NMR (DMSO-d6): 179.19 (C-16); 169.87 (C-1); 156.94 (C-9); 139.33 (Cq); 135.22 (Cq); 133.16 (Cq); 131.04; 130.43; 129.14 (C-11, C-13); 128.93; 128.48; 128.35; 127.83; 126.82; 124.65 (C-12); 123.48; 120.84 (C-19); 116.37 (C-10, C-14); 67.72 (C-8). FT-IR (solid in ATR, ν cm^−1^): 3220m; 3106w; 3058m; 3035m; 1666m; 1582m; 1526vs; 1491vs; 1477s; 1437m; 1410m; 1323m; 1292m; 1238vs; 1162s; 1091m; 1028s; 1005m; 816m; 772m; 706m; 678m; 658w. Anal. Calcd for C_21_H_16_BrClN_2_O_2_S (475.78): C, 53.01; H, 3.39; Br, 16.79; Cl, 7.45; N, 5.89; S, 6.74%; Found: C, 53.37; H, 3.25; Br, 16.83; Cl, 7.36; N, 6.45; S, 6.76%.

*2-((4-Chlorophenoxy)methyl)-N-(2,6-dibromophenylcarbamothioyl)benzamide* (**1h**). Yield 43%; mp 200.4–202.2 °C; ^1^H-NMR (DMSO-d6): 12.12 (br s, 1H, NH, deuterable); 11.93 (br s, 1H, NH, deuterable); 7.72 (d, *J* = 8.0 Hz, 2H, H-19, H-21); 7.64 (bd, *J* = 7.2 Hz, 1H, H-7); 7.59 (bd, *J* = 7.5 Hz, 1H, H-4); 7.57 (td, *J* = 1.4 Hz, *J* = 7.5 Hz, 1H, H-5); 7.48 (td, *J* = 1.4 Hz, *J* = 7.5 Hz, 1H, H-6); 7.31 (d, *J* = 9.0 Hz, 2H, H-11, H-13); 7.21 (t, *J* = 8.0 Hz, 1H, H-20); 7.01 (d, *J* = 9.0 Hz, 2H, H-10, H-14); 5.29 (s, 2H, H-8). ^13^C-NMR (DMSO-d6): 180.52 (C-16); 169.79 (C-1); 156.92 (C-9); 138.69 (Cq); 136.69 (Cq); 134.97 (Cq); 133.33 (Cq); 132.07 (C-19, C-21); 131.08; 130.42; 129.12 (C-11, C-13); 128.56; 128.07; 124.52 (C-12); 123.97 (C-18, C-22); 116.44 (C-10, C-14); 67.65 (C-8). FT-IR (solid in ATR, ν cm^−1^): 3155s; 3003m; 2935w; 2861w; 1686s; 1504vs; 1488vs; 1459s; 1430m; 1388m; 1346m; 1260m; 1237s; 1171s; 1153m; 1104m; 1078m; 1050w; 1029m; 1005m; 825m; 772m; 746m; 728m; 675w; 654m. Anal. Calcd for C_21_H_15_Br_2_ClN_2_O_2_S (554.68): C, 45.47; H, 2.73; Br, 28.81; Cl, 6.39; N, 5.05; S, 5.78%; Found: C, 45.29; H, 2.89; Br, 28.98; Cl, 6.31; N, 6.11; S, 5.66%.

*2-((4-Chlorophenoxy)methyl)-N-(2-iodophenylcarbamothioyl)benzamide* (**1i**). Yield 78%; mp 152.7–154.2 °C; ^1^H-NMR (DMSO-d6): 12.22 (br s, 1H, NH, deuterable); 12.03 (br s, 1H, NH, deuterable); 7.92 (dd, *J* = 1.4 Hz, *J* = 8.0 Hz, 1H, H-19); 7.65 (bd, *J* = 7.4 Hz, 1H, H-7); 7.62–7.55 (m, 3H, H-4, H-5, H-22); 7.50 (td, *J* = 7.5 Hz, 1H, H-6, 1.4); 7.44 (td, *J* = 7.6 Hz, , *J* = 1.4 Hz, 1H, H-21); 7.32 (d, *J* = 9.0 Hz, 2H, H-11, H-13); 7.08 (td, *J* = 7.6 Hz, *J* = 1.4 Hz, 1H, H-20); 7.02 (d, *J* = 9.0 Hz, 2H, H-10, H-14); 5.32 (s, 2H, H-8). ^13^C-NMR (DMSO-d6): 180.24 (C-16); 170.08 (C-1); 156.93 (C-9); 140.05 (Cq); 138.79 (Cq); 135.10 (Cq); 133.23 (Cq); 131.08; 129.19 (C-11, C-13); 128.78; 128.69; 128.67; 128.39; 127.94; 124.59 (C-12); 116.44 (C-10, C-14); 129.86; 96.96 (C-18); 67.68 (C-8). FT-IR (solid in ATR, ν cm^−1^): 3220m; 3026w; 1674m; 1571w; 1525vs; 1490vs;1434m; 1389m; 1320m; 1278m; 1242vs; 1162s; 1093w; 1037m; 1016w; 815m; 745m; 712m; 683m; 662w. Anal. Calcd for C_21_H_16_ClIN_2_O_2_S (522.78): C, 48.25; H, 3.08; Cl, 6.78; I 24.27; N, 5.36; S, 6.13%; Found: C, 48.49; H, 2.96; Cl, 6.61; I, 24.39; N, 5.29; S, 6.05%.

*2-((4-Chlorophenoxy)methyl)-N-(4-iodophenylcarbamothioyl)benzamide* (**1j**). Yield 53%; mp 154.3–156.2 °C; ^1^H-NMR (DMSO-d6): 12.37 (br s, 1H, NH, deuterable); 11.88 (br s, 1H, NH, deuterable); 7.75 (d, *J* = 8.8 Hz, 2H, H-18, H-22); 7.62 (bd, *J* = 7.4 Hz, 1H, H-7); 7.59 (bd, *J* = 7.4 Hz, 1H, H-4); 7.57 (td, *J* = 1.4 Hz, *J* = 7.5 Hz, 1H, H-5); 7.47 (td, *J* = 1.4 Hz, *J* = 7.5 Hz, 1H, H-6); 7.43 (d, *J* = 8.8 Hz, 2H, H-19, H-21); 7.31 (d, *J* = 9.0 Hz, 2H, H-11, H-13); 7.00 (d, *J* = 9.0 Hz, 2H, H-10, H-14 ); 5.31 (s, 2H, H-8). ^13^C-NMR (DMSO-d6): 178.85 (C-16); 169.86 (C-1); 156.92 (C-9); 137.62 (Cq); 137.22 (C-19, C-21); 135.20 (Cq); 133.14 (Cq); 131.03; 129.15 (C-11, C-13); 128.50; 128.32; 127.80; 126.34 (C-18, C-22); 124.62 (C-12); 116.38 (C-10, C-14); 91.00 (C-20); 67.68 (C-8). FT-IR (solid in ATR, ν cm^−1^): 3347s; 3156w; 3326m; 2907w; 1676m; 1595m; 1581m; 1547m; 1511s; 1486vs; 1443s; 1396m; 1330m; 1287m; 1239s; 1148s; 1030m; 1001m; 943w; 855w; 817m; 755m; 751m; 738m; 699w; 684w; 650m. Anal. Calcd for C_21_H_16_ClIN_2_O_2_S (522.78): C, 48.25; H, 3.08; Cl, 6.78; I 24.27; N, 5.36; S, 6.13%; Found: C, 48.09; H, 3.17; Cl, 6.89; I, 24.36; N, 5.34; S, 6.19%.

*2-((4-Chlorophenoxy)methyl)-N-(3-nitrophenylcarbamothioyl)benzamide* (**1k**). Yield 59%; mp 165.6–167.4 °C; ^1^H-NMR (DMSO-d6): 12.53 (br s, 1H, NH, deuterable); 12.03 (br s, 1H, NH, deuterable); 8.66 (t, *J* = 2.2 Hz, 1H, H-18); 8.12 (ddd, *J* =1.0 Hz, *J* =2.2 Hz, *J* = 8.3 Hz, 1H, H-20); 7.94 (ddd, *J* = 1.0 Hz, *J* = 2.2 Hz, *J* = 8.3 Hz, 1H, H-22); 7.69 (t, *J* = 8.3 Hz, 1H, H-21); 7.65 (bd, *J* = 7.4 Hz, 1H, H-7); 7.60 (bd, *J* = 7.4 Hz, 1H, H-4); 7.59 (td, *J* = 1.4 Hz, *J* = 7.5 Hz, 1H, H-5); 7.49 (td, *J* = 1.4 Hz, *J* = 7.5 Hz, 1H, H-6); 7.30 (d, *J* = 9.0 Hz, 2H, H-11, H-13); 7.02 (d, *J* = 9.0 Hz, 2H, H-10, H-14 ); 5.32 (s, 2H, H-8). ^13^C-NMR (DMSO-d6): 179.54 (C-16); 169.80 (C-1); 156.94 (C-9); 147.41 (C-19); 139.00 (Cq); 135.27 (Cq); 133.14 (Cq); 131.10; 131.00; 129.86; 129.15 (C-11, C-13); 128.51; 128.37; 127.85; 124.67 (C-12); 120.78; 118.94; 116.37 (C-10, C-14); 67.71 (C-8). FT-IR (solid in ATR, ν cm^−1^): 3164m; 3083w; 2929w; 1685m; 1584vs; 1490s;1386w; 1344m; 1313w; 1278m; 1246m; 1230m; 1174m; 1151m; 1091w; 1070w; 1023m; 1003w;819m; 767w; 740m; 698w; 678w. Anal. Calcd for C_21_H_16_ClN_3_O_4_S (441.88): C, 57.08; H, 3.65; Cl, 8.02; N, 9.51; S, 7.26%; Found: C, 57.32; H, 3.57; Cl, 7.89; N, 9.56; S, 7.28%.

*2-((4-Chlorophenoxy)methyl)-N-(4-nitrophenylcarbamothioyl)benzamide* (**1l**). Yield 68%; mp 173.5–175.2 °C; ^1^H-NMR (DMSO-d6): 12.69 (br s, 1H, NH, deuterable); 12.05 (br s, 1H, NH, deuterable); 8.27 (d, *J* = 9.2 Hz, 2H, H-19, H-21); 8.00 (d, *J* = 9.2 Hz, 2H, H-18, H-22); 7.65 (bd, *J* = 7.4 Hz, 1H, H-7); 7.60 (bd, *J* = 7.4 Hz, 1H, H-4); 7.59 (td, *J* = 1.4 Hz, *J* = 7.5 Hz, 1H, H-5); 7.49 (td, *J* = 1.4 Hz, *J* = 7.5 Hz, 1H, H-6); 7.30 (d, *J* =9.0 Hz, 2H, H-11, H-13); 7.01 (d, *J* = 9.0 Hz, 2H, H-10, H-14); 5.32 (s, 2H, H-8). ^13^C-NMR (DMSO-d6): 178.96 (C-16); 169.81 (C-1); 156.90 (C-9); 144.23 (C-20); 143.78 (C-17); 135.31 (Cq); 133.00 (Cq); 131.15; 129.16 (C-11, C-13); 128.56; 128.35; 127.82; 124.18 (C-18, C-22); 123.89 (C-19, C-21); 124.61 (C-12); 116.35 (C-10, C-14); 67.63 (C-8). FT-IR (solid in ATR, ν cm^−1^): 3352m; 3078w; 2974m; 1678m; 1566m; 1513s; 1490vs; 1445m; 1332m; 1299s; 1262m; 1242vs; 1145s; 1111m; 1029m; 960w; 863m; 846m; 820w; 781w; 743m; 699w; 680w; 652m. Anal. Calcd for C_21_H_16_ClN_3_O_4_S (441.88): C, 57.08; H, 3.65; Cl, 8.02; N, 9.51; S, 7.26%; Found: C, 56.82; H, 3.77; Cl, 8.14; N, 9.59; S, 7.26%.

#### 4.2.2. Synthesis and Characterization of Core@Shell Nanostructure

Core@shell—Fe_3_O_4_@C_18_ nanostructure was prepared and characterized in the same manner to our previously published papers that report the successful fabrication of Fe_3_O_4_@C_14_ and Fe_3_O_4_@C_18_ [27,35]. Briefly, stearic acid (C_18_) was dispersed in 200 mL volume of distilled-deionized water, corresponding to a 0.50% (*v*/*w*) solution, under vigorous stirring at 60 °C. Five mL solution consisting of 25% NH_3_ was added to C_18_ dispersion. Fe^2+^:Fe^3+^ (1:2 molar ratio) were dropped under permanent stirring in aqueous solution of NH_3_, leading to the formation of a black precipitate. The product was repeatedly washed with methanol and separated with a strong NdFeB permanent magnet.

The successful fabrication of Fe_3_O_4_@C_18_ nanostructure was confirmed by XRD, TEM and FT-IR.

X-ray diffraction analysis was performed on a XRD 6000 diffractometer (Shimadzu Tokyo 101-8448, Chiyoda-ku, Japan) at room temperature. In all the cases, Cu Kα radiation from a Cu X-ray tube (run at 15 mA and 30 kV) was used. The samples were scanned in the Bragg angle 2θ range of 10–80°.

TEM images were obtained on finely powdered samples using a Tecnai™ G2 F30 S-TWIN high-resolution transmission electron microscope from FEI Company (Hillsboro, OR, USA). The microscope was operated in transmission mode at 300 kV with TEM point resolution of 2 Å and line resolution of 1 Å. The fine hybrid nanostructure was dispersed into pure ethanol and ultrasonicated for 15 min. After that, diluted sample was put onto a holey carbon-coated copper grid and left to dry before TEM analysis.

A Nicolet 6700 FT-IR spectrometer (Thermo Nicolet, Madison, WI, USA), connected to the OMNIC operating system software (Version 7.0 Thermo Nicolet, Waltham, MA, USA) was used to obtain FT-IR spectra of hybrid materials. The samples were placed in contact with attenuated total reflectance (ATR) on a multibounce plate of ZnSe crystal at controlled ambient temperature (25 °C). FT-IR spectra were collected at a resolution of 4 cm^−1^ with strong apodization, in the frequency range of 4000–650 cm^−1^ by co-adding 32 scans. All spectra were ratioed against a background of an air spectrum.

#### 4.2.3. Fabrication of Coverslips Coated with Core@Shell@Adsorption-Shell Nanostructure

The adsorption-shell represented by 15 mg of (**1a–l**) was solubilized in 1 mL of chloroform together with 135 mg Fe_3_O_4_@C_18_ and grounding until complete evaporation of chloroform. This step is repeated by three times for uniform distribution of the **1a–l** on the surface of spherical nanostructure. The fabrication was performed by coating the coverslips with nanofluid represented by suspended core@shell@adsorption-shell in chloroform (0.33% *w*/*v*) according to our published papers [27]. The coated coverslips were then sterilized by ultraviolet irradiation for 15 min.

### 4.3. Microbial Strains Used for Antimicrobial Activity Assay

The influence of the obtained functionalized surfaces on the fungal biofilms growth was carried using fungal suspensions of 0.5 McFarland obtained from 24 h cultures [36], using *C. albicans* ATCC10231 reference strain.

### 4.4. Microbiological Assay Investigation Procedure—Microbial Adherence to the Coated Slide Specimens

The microbial adherence ability was investigated in six multiwell plates, in which there have been placed 1 cm^2^ slides either (i) coated with the chemical compound, using as negative control the glass slide as well and respectively; (ii) magnetic nanoparticles loaded with the tested substances, using as negative control the glass slide covered with nanoparticles. Plastic wells were filled with 2 mL liquid medium, inoculated with 300 μL 0.5 McFarland fungal suspensions and incubated for 24 h at 37 °C. After 24 h, the culture medium was removed, the slides were washed three times in phosphate buffered saline (PBS) in order to remove the non-adherent strains and fresh glucose broth was added, the same procedure as mentioned above being repeated after 48 h incubation period. For each 24 h, viable cell counts have been achieved for both working variants in order to assess the biofilm forming ability of the two strains. To this purpose, the adhered cells have been removed from the slides by vortexing and brief sonication and serial dilutions ranging from 10^−1^ to 10^−16^ of the obtained inocula have been spotted on Muller Hinton agar, incubated for 24 h at 37 °C and assessed for viable cell counts.

### 4.5. Biocompatibility

Human umbilical cords were obtained under sterile conditions after birth from full-term infants, with the consent of the parents. The umbilical cord blood vessels (two arteries and one vein) were removed and the remaining tissue was diced into small fragments (1 mm^3^ pieces) and treated with 2 mg/mL collagenase II for 3–4 h at 37 °C. Cell suspension was centrifuged, washed and resuspended in α-minimal essential medium (GIBCO, Gaithersburg, MD, USA) supplemented with 10% fetal bovine serum (GIBCO, Gaithersburg, MD, USA) and 10 ng/mL basic fibroblast growth factor (bFGF) (Sigma, St. Louis, MO, USA). The cells were plated at a density of 1 × 10^4^ cells/cm^2^ and cultured at 37 °C in a humidified atmosphere containing 5% CO_2_. The medium was changed every 3 days and passaged by trypsinization when the cells reached 70–80% confluence. Cells at the passage 14 of were used in our experiments. 2 × 10^5^ cells were seeded on the functionalized slides distributed in 6 wells plate. 24 h later, the cells were fixed in ethanol 70% and stained with 50 µg/mL propidium iodide. The morphology of lived or stained cells were observed using Observer.D1 inverted microscope (Carl Zeiss AG, Dublin, CA, USA).

## 5. Conclusions

We have synthesized new 2-((4-chlorophenoxy)methyl)-*N*-(substituted phenylcarbamo-thioyl)benzamide by an efficient method. The structure of the prepared compounds was determined by spectral studies and elemental analysis. The antibiofilm effect of the obtained compounds was dependent on the position and nature of the substituents, demonstrating that structural modulation could be very useful to enhance the antimicrobial properties of thiourea analogs but also their interaction with potential carriers. The combinations of the tested compounds with fabricated Fe_3_O_4_@C_18_ re*s*ulted in increased efficiency in preventing the in vitro development of *C. albicans* biofilms, suggesting that the obtained hybrid nanosystems could represent an effective, biocompatible strategy with prophylactic and therapeutic value in fighting biofilm associated *C. albicans* infections.
